# Deep into Cognition: The Neuropsychological Identikit of Younger and Older Individuals after COVID-19 Infection

**DOI:** 10.3390/biology13100754

**Published:** 2024-09-24

**Authors:** Maria Devita, Adele Ravelli, Anna Panzeri, Elisa Di Rosa, Pamela Iannizzi, Gioia Bottesi, Chiara Ceolin, Marina De Rui, Annamaria Cattelan, Silvia Cavinato, Chiara Begliomini, Biancarosa Volpe, Rossana Schiavo, Marta Ghisi, Daniela Mapelli

**Affiliations:** 1 Department of General Psychology, University of Padova, 35131 Padova, Italy; maria.devita@unipd.it (M.D.); anna.panzeri@unipd.it (A.P.); elisa.dirosa@unipd.it (E.D.R.); gioia.bottesi@unipd.it (G.B.); chiara.begliomini@unipd.it (C.B.); biancarosa.volpe@unipd.it (B.V.); marta.ghisi@unipd.it (M.G.); daniela.mapelli@unipd.it (D.M.); 2 Geriatrics Unit, Department of Medicine, University of Padua, 35128 Padova, Italy; chiara.ceolin.1@phd.unipd.it (C.C.); marina.derui@aopd.veneto.it (M.D.R.); 3 Veneto Institute of Oncology IOV IRCCS Padua, 35128 Padova, Italy; pamela.iannizzi@iov.veneto.it; 4 Infectious and Tropical Diseases Unit, Padua University Hospital, 35128 Padova, Italy; annamaria.cattelan@aopd.veneto.it (A.C.); silvia.cavinato@aopd.veneto.it (S.C.); 5 Padua Neuroscience Center, University of Padova, 35131 Padova, Italy; 6 Hospital Psychology Unit, Padua University Hospital, 35128 Padova, Italy; rossana.schiavo@aopd.veneto.it

**Keywords:** neurocovid, COVID-19, brain fog, younger adults, memory frailty

## Abstract

**Simple Summary:**

This study addresses the ongoing scientific debate regarding the existence, causes, characteristics and reversibility of cognitive sequelae associated with COVID-19 infection. The aim of the study is to describe the neuropsychological profile of individuals diagnosed with COVID-19, distinguishing between younger (<65 years) and older (≥65 years) adults and evaluating them at baseline and at 3 and 6 months after infection. Significant differences in cognitive performance between younger and older adults are observed, consistent with their different physiological conditions. However, distinct memory recall patterns not attributable to physiological differences are also observed. The results contribute to the understanding of COVID-19 cognitive sequelae, suggesting that cognitive deficits in COVID-19 survivors may primarily reflect difficulties in attention and concentration impacting retrieval processes, aligning with the concept of “brain fog” associated with post-COVID-19 syndrome.

**Abstract:**

The literature on COVID-19 continues to increase daily. Cognitive sequelae associated with COVID-19 infection still draw the attention of the scientific community given the lack of consensus about their existence, etiology, characterization and reversibility. The aim of this study is to provide a neuropsychological identikit for younger (<65 years) and older (≥65 years) individuals diagnosed with COVID-19 infection, at baseline and after 3 and 6 months. In total, 226 individuals took part in a retrospective observational study and their cognitive performance was compared across groups (younger adults vs. older adults) and time (T0, T1, T2). The results highlighted differences between younger and older adults in the Montreal Cognitive Assessment (MoCA) global score, as expected in consideration of the different physiological conditions of the two populations. However, memory performance highlighted the two groups as characterized by a difference in patterns of recall that may move beyond a physiological explanation and provide information about COVID-19 cognitive sequelae. This study suggests that cognitive deficits observed in COVID-19 survivors may reflect a difficulty in attention and concentration that interferes mainly with retrieval processes. This result fits well with the concept of “brain fog” typical of post-COVID-19 syndrome and may also reflect the stress experienced while facing the pandemic.

## 1. Introduction

Although the literature on COVID-19 is comprehensive, some issues remain open and deserve to be more deeply examined. Among these, cognitive sequelae associated with COVID-19 infection still draw the attention of the scientific community given the lack of consensus about their actual existence, etiology, characterization and reversibility. In the specific scientific literature, and as reported by individuals after the acute phase of infection, these neuropsychological symptoms are often described as “brain fog”. However, it has been suggested that these cognitive sequelae could go beyond the classical post-viral syndrome (commonly characterized by mental and physical exhaustion and depression [1]), since they were found to be not associated with fatigue and mood symptoms, thus being a specific post-COVID-19 manifestation [2].

Several efforts have been made to investigate, even longitudinally [3,4], the cognitive sequelae associated with COVID-19, both during the acute phase of infection [5,6] and after its resolution [7,8]. In general, although there is a consensus that a proportion of individuals show quantifiable cognitive frailty at least several months after the infection, some discrepancies can be found concerning the constellation of the cognitive domains involved. While some studies have shown multi-domain involvement, describing deficits mainly attributable to executive function [9], memory, attention and concentration [2], the speed of processing and fluency [10], some others support a more specific and isolated deficit [11]. Other evidence, finally, does not report any significant differences between healthy controls who do not have COVID-19 and COVID-19 individuals [12]. Another issue concerns the evolution of these deficits over time. The main pattern that stands out from the literature is that of partial reversibility, so that a significant improvement can be observed from 6 months after the infection, despite some deficits remaining at least in this span of time [3,4]. However, the literature still lacks studies that clarify the relationship between COVID-19 and cognitive impairment, particularly regarding the characterization of the specific affected domains and their evolution over time [13]. In concert with these issues, what needs to be further explored is the variables that can account for the differences observed in the neuropsychological involvement among COVID-19 symptoms. For example, old age, comorbidities, the intensity of the treatment received and the severity of the infection appear to be determining factors in the prognostic outcome [13,14]. Old age and comorbidities not only represent prominent risk factors for severe illness, but are also a relevant aspect influencing the cognitive status of individuals recovering from COVID-19 [5,15,16]. In fact, this population largely overlaps with that most at risk of developing neurocognitive disorders. In some cases, indeed, the virus undermines a pre-existing condition of neurocognitive frailty that can increase the susceptibility to cognitive consequences due to inflammatory states [17]. While it is true that several studies agree that the increase in age correlates with the severity of cognitive dysfunction, efforts to explore the specific differences between young and older adults are still only preliminary. Specifically, to the best of our knowledge, no studies have explored in depth whether any differences concerning the specific affected cognitive domains exist between younger and older adults or examined their possible differences in evolution over time. A plausible hypothesis that can be put forth is that the infection might exhibit distinct effects and courses within cohorts of younger and older adults, owing to the increased fragility and diminished resilience typical of the latter group [18]. A further relevant aspect emerges from the fact that, while the common concern of “brain fog” refers to the inability to concentrate and poor attention, when cognitive impairment is present, memory complaints are the most commonly reported [19,20]. A deeper insight into the characteristics of memory performance will help to disentangle what specific processes are mainly involved in younger and older adults and, specifically, if the difficulties experienced reflect a genuine memory deficit or rather frailty in the attentional–executive processes involved in the spontaneous recall of information. Thus, the present paper aims to contribute to the literature by providing a neuropsychological identikit for young and old individuals after COVID-19 infection, at baseline and at 3 and 6 months after the infection, in order to raise awareness about the nature and the possible differences in the evolution of cognitive sequelae affecting young and old COVID-19 survivors.

## 2. Materials and Methods

This is a retrospective observational study based on a broader project involving the Hospital Operational Units of Psychology, Infectious Diseases and Pneumology. The overall project, through the collection of a large amount of data, aims to contribute to the framing of the cognitive and psychological profiles of individuals diagnosed with COVID-19 since the first wave and to evaluate their evolution over time [15,21]. All participants had previously recovered at the Infectious Diseases Unit because of complications due to COVID-19 infection.

The inclusion criteria were previous positivity to SARS-CoV-2, aged from 18 to 90 years, previous hospitalization and subsequent access to outpatient follow-up at the Infectious and Tropical Diseases Unit. The exclusion criteria were being non-native Italian speakers and suffering from sensory and/or motor limitations that prevented psychometric testing. Additionally, individuals with a history of neurological and/or psychiatric conditions, whether recent or remote, that might affect their cognitive performance on the tests were also excluded from the study. This information was gathered during the anamnesis through a semi-structured interview. All participants provided written informed consent before entering the study. The whole sample was sorted into younger adults (<65 years) and older adults (≥65 years), according to previous authors [22,23].

Data were collected at three time points, during follow-up clinical visits at the Infectious and Tropical Diseases Unit. The first follow-up was carried out within one month after discharge, where participants underwent cognitive and psychological examinations; moreover, information regarding sociodemographic characteristics, proximate pathological anamnesis due to COVID-19, remote pathological anamnesis and general lifestyle habits was obtained retrospectively from medical records and the semi-structured interview mentioned above. The COVID-19 severity was operationalized taking into account the intensity of the care received. Specifically, from the least to the most severe levels, this included no oxygen supply (AA), a high-flow nasal cannula (HFNC), non-invasive ventilation (NIV) and intensive oxygen therapy (IOT).

To obtain a brief and sensitive measure of global cognition, the Montreal Cognitive Assessment (MoCA [24]) was administered. The MoCA evaluates global cognitive functioning through items assessing short-term memory; visuospatial abilities by clock drawing and a cube copy task; executive functioning through an adaptation of the Trail Making Test Part B, phonemic fluency and verbal abstraction; attention, concentration and working memory by means of target detection, serial subtraction, digits and digits backward; language via confrontation naming with low-familiarity animals and the repetition of complex sentences; and orientation to time and place. The maximum achievable score is 30, with a higher score indicative of better cognitive functioning.

For the purpose of this sub-study, 202 participants (115 females; mean age 56.8 ± 14 years; range 18–88 years; mean education 12.6 ± 4.5 years; range 2–25 years) were consecutively enrolled. Of the whole initial sample, 137 participants returned for the second follow-up (T1) and 79 for the last follow-up (T2). The second and third follow-ups occurred, respectively, three and six months after hospital discharge. The same psychometric tools used in the first assessment were administered.

Descriptive demographic, clinical and neuropsychological characteristics of the sample as a whole and sorted by age are provided. Continuous variables are expressed as the mean and standard deviation (M ± SD); categorical variables are expressed as numerosity and frequencies (n, %), where appropriate. Two-sample independent *t*-Student’s tests were carried out comparing younger adults (YA) and older adults (OA); a repeated-measures ANOVA (2 × 3) was also performed, considering AGE (YA vs. OA) as the between and TIME (T0, T1, T2) as the within factor. To account for potential confounders, an ANCOVA was carried out in the cross-sectional analyses to assess differences between YA and OA, adjusting for biological sex, education level, comorbidities and COVID-19 severity. Furthermore, a repeated-measures ANOVA with a multivariate model for follow-up tests was used to examine the effect of AGE over time (between groups) and the influence of the aforementioned variables (within subjects). Statistical analyses were carried out using the JASP 0.17 (Intel) software (https://jasp-stats.org (accessed on 18 September 2023)).

## 3. Results

The descriptive demographic, clinical and neuropsychological characteristics of the sample as a whole and sorted by age are summarized in Table 1.

As reported, the mean age of the participants was 56.78 ±14.03 and the sample was gender-balanced. In most cases, the COVID-19 severity was mild, with 36.1% of the total sample requiring no oxygen support and 39.6% needing an oxygen mask. The most frequent comorbidity was cardiovascular disease, particularly among older adults (56.9%), followed by dyslipidemia (28.7% of the total sample).

### 3.1. Cross-Sectional Analyses

Significantly higher scores in the MoCA test (i.e., higher cognitive functioning) were observed in YA compared to OA (t(174) = 6.03, *p* < 0.01, d’Cohen: 0.98). Significant differences emerged between the groups also for the TMT-B (t(171) = 2.08, *p* < 0.05, d’Cohen: 0.34), drawing copy (t(171) = 4.49, *p* < 0.01, d’Cohen: 0.73), clock test (t(171) = 3.44, *p* < 0.01, d’Cohen: 0.56), naming (t(171) = 2.09, *p* < 0.05, d’Cohen: 0.34), digit span (t(173) = 3.39, *p* < 0.01, d’Cohen: 0.55), phonetic fluency (t(173) = 2.35, *p* < 0.05, 0.38), abstraction (t(173) = 3.28, *p* < 0.01, d’Cohen: 0.53), free recall (t(173) = 4.41, *p* < 0.01, d’Cohen: 0.71) and recall with semantic cue (t(172) = −2.28, *p* < 0.05, d’Cohen: 0.37) subtests. The ANCOVA model, adjusted for potential confounders, confirmed the significant effect of age (YA vs. OA) on the baseline MoCA global scores (F(1, 200) = 13.491, *p* < 0.001), as well as education (F(1, 200) = 30.419, *p* < 0.001). In contrast, no significant effects were observed for biological sex, comorbidities or the COVID-19 severity on the MoCA scores.

### 3.2. Longitudinal Analyses

**Figure 1:**
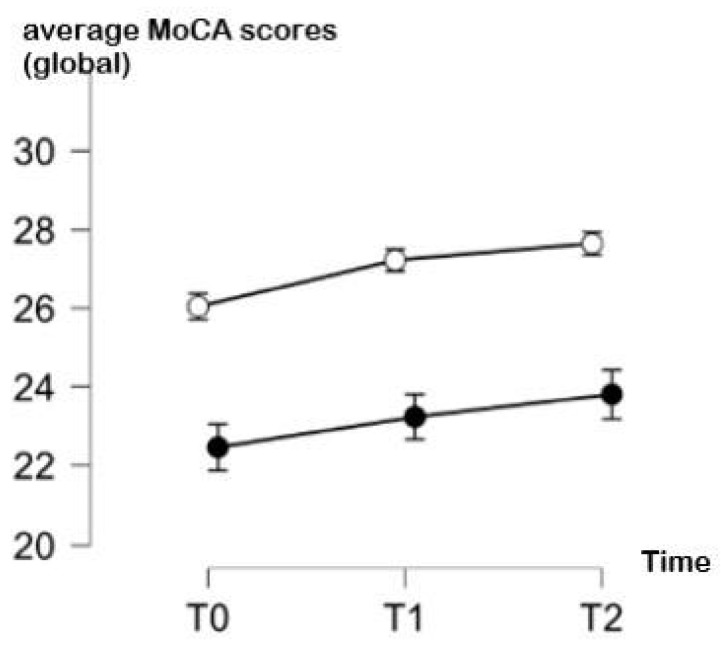
Average MoCA scores (and standard deviations) over time (T0: 1 month, T1: 3 months, T2: 6 months since infection) in young adults (white points) and in older adults (black points).

The comparison of the cognitive performance of YA and OA over time in the global MoCA test, adjusted for education and the severity of COVID-19, suggests that both groups showed a continuous improvement across the three time points of evaluation. However, the YA showed a more evident improvement between T0 and T1 (respective means ± SD: 26.1 ± 2.1 vs. 27.2 ± 2.3) and the stabilization of their performance between T1 and T2 (respective means ± SD: 27.2 ± 2.3 vs. 27.5 ± 2.09). Interestingly, OA showed an improvement in the MoCA global score also between T1 and T2 (respective means ± SD: 23.5 ± 4.4 and 23.8 ± 4.3) beyond T0 and T1 (respective means ± SD: 21.7 ± 4.9 and 23.5± 4.44). Complete data are shown in Table 2, and a graphical representation is provided in Figure 1.

As for the repeated-measures ANOVA, a significant between-factor main effect of age was observed (F(1, 200) = 13.682, *p* < 0.001), with OA performing significantly poorer than YA. Education also emerged as a significant factor across time (F(1, 200) = 20.355, *p* < 0.001). Biological sex approached significance (F(1, 200) = 4.063, *p* = 0.049), suggesting a potential trend worth further exploration. No significant effects were found for other between-subject variables, such as comorbidities or the COVID-19 severity. As for within-subject effects, no significant differences were detected (F(2, 400) = 0.675, *p* = 0.511). Additionally, none of the interaction effects between time and the between-subject factors (age, education, sex, comorbidities and COVID-19 severity) reached statistical significance.

Looking at the MoCA subtests and, in particular, at the clock test over time, YA were generally steady in the three evaluations (respective means ± SD: 2.9 ± 0.6 vs. 2.8 ± 0.5 vs. 2.8 ± 0.4); moreover, OA showed steady performance between T0 and T1, as well as YA (respective means ± SD 2.3 ± 0.9 vs. 2.3 ± 1). In contrast, a slight improvement in OA could be observed between T1 and T2 (respective means ± SD 2.3 ± 1 vs. 2.4 ± 0.8). Furthermore, a significant effect of the COVID-19 severity was observed as a within-subjects effect (F(2) = 5.312, *p* = 0.006).

As for the memory subtest and, in particular, for the categorical delayed recall item, YA showed steady performance between T0 and T1 (respective means ± SD: 0.7 ± 0.9 vs. 0.7 ± 0.9), while a decrease could be observed between T1 and T2 (respective means ± SD: 0.7 ± 0.9 vs. 0.5 ± 0.7). Meanwhile, OA showed a significant decrease in their categorical recall between T0 and T1 (respective means ± SD: 1.5 ± 1.1 vs. 0.8 ± 0.9) and still a slight decrease also between T1 and T2 (respective means ± SD: 0.8 ± 0.9 vs. 0.7 ± 0.6). Furthermore, a significant effect of age was observed as a within-subjects effect (F(2) = 3.842, *p* = 0.024).

**Figure 2:**
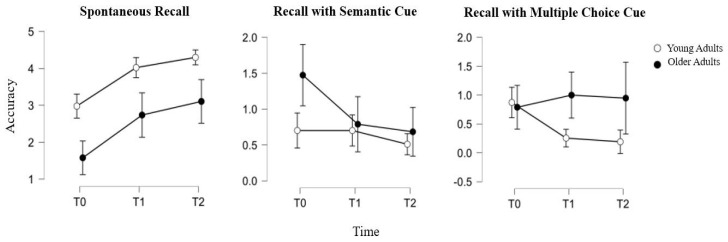
Accuracy in memory indices of MoCA over time (T0: 1 month, T1: 3 months, T2: 6 months since infection) in young adults (white points) and in older adults (black points).

Moreover, for the multiple-choice item of memory recall, differences between YA and OA emerged. In particular, YA showed a constant decrease in their performance, with a more significant decline between T0 and T1 (respective means ± SD: 0.9 ± 1 vs. 0.3 ± 0.5) than between T1 and T2 (respective means ± SD: 0.3 ± 0.5 vs. 0.2 ± 0.5). OA, instead, showed a slight improvement between T0 and T1 (respective means ± SD: 0.8 ± 1.1 vs. 1 ± 1.2) and almost steady performance between T1 and T2 (respective means ± SD: 1 ± 1.2 vs. 0.9 ± 1.1). Furthermore, a significant effect of age was observed as a within-subjects effect (F(2) = 4.066, *p* = 0.019). A graphical representation of the performance in the memory subtests of the MoCA of the YA and OA over time is provided in Figure 2.

## 4. Discussion

This paper contributes to the longitudinal exploration of cognition in individuals that have suffered from COVID-19 infection by focusing on certain, specific factors (i.e., age differences). As previously shown, age-related differences in facing the pandemic have been described [15]. These findings, as with others provided by the literature [25,26], although potentially interesting, lack longitudinal evidence, beyond being limited to a global observation of neuropsychological functioning, without specific attention to or a particular focus on the single subtests aimed at measuring different cognitive domains. Instead, in this paper, a deeper cognitive “identikit” for young and older adults after COVID-19 infection is provided and described in a period of six months. Although differences between young and older adults in cognitive performance are expected, if not even foregone, our results seem to innovatively increase the current literature about COVID-19. As a matter of fact, our findings show different, interesting patterns of cognitive performance that are, in some aspects, counterintuitive.

On the one hand, the global scores obtained in the MoCA test are absolutely in line with what is expected physiologically: YA show higher scores than OA and the improvement over time is expected to be slighter. This result may be interpreted as a ceiling performance effect: YA do not show any particular cognitive frailty (also considering that the MoCA test can be perceived as simple for this population) and their potential improvement can be only slight. In contrast, OA may have more difficulty in performing a cognitive test for the first time and show an increased impact of COVID-19’s effects at the very first time after infection. However, they show increased performance over time, supporting the hypothesis of the reversibility of the deficits associated with COVID-19 [3] and suggesting that recovery is still possible despite the first signs of cognitive frailty.

Furthermore, delving deeper into cognition, interesting results have been found, especially for the memory subtest. As can be seen, the spontaneous recall follows the trend of the global score described above, progressively improving in both groups, with a physiological age-related difference. A deeper insight into YA and OA mnestic frailty can be achieved through a careful look at the cues used to facilitate delayed recall, namely the semantic cue and multiple-choice recognition. Given a missed spontaneous recall, effective cued recall reflects an apparent memory deficit that is secondary to reduced attentional resources or ineffective recall strategies [27]. Otherwise, when it is necessary to resort to multiple-choice recognition, frailty in specific memory processes such as encoding and retrieval is more likely [28].

At T0, YA make more use of multiple-choice recognition. Given the marginality of the spontaneous recall deficit and its reversibility, in addition to the age of the sample, it is unlikely that this is due to a genuine episodic memory deficit. Most likely, this temporary vulnerability close to infection is attributable to a decreased processing capacity affecting the learning phase, as stated in the Introduction section, which can be interpreted in light of the “brain fog” widely described in the literature [19,20] or the stress experienced during the pandemic and the confinement period, consistent with the findings reported by Devita et al. (2021) [15]. Over time, in fact, the almost ceiling improvement corresponds to almost no use of semantic or multiple-choice clues. On the other hand, in the case of OA, the semantic cue is predominantly used at T0, much more widely than in the case of YA. In the two following evaluations, the use of semantic cues decreases markedly, and, at the same time, the use of multiple-choice recognition increases. This opposite trend in the effectiveness of cues suggests that, as well as YA, OA experienced executive–attentive difficulties, so that the recall improved significantly with semantic cueing. Over time, alongside the improvement in spontaneous recall, the remaining difficulties were no longer adequately compensated for by semantic cueing, instead requiring multiple-choice recognition. It is therefore plausible that the remaining difficulties reflect forgetfulness due to a failure to use effective processing strategies or other factors that limit learning, typical of the aging process [28] and independent of the sequelae of COVID-19, progressively attenuated.

Somewhat unexpectedly compared to the existing literature [29], in this study, differences between males and females did not emerge, at least concerning the cognitive variables. Further research on the topic is undoubtedly needed to better understand the sex differences related to COVID-19 infection. Interestingly, our results seem not to be influenced by other factors, such as comorbidities (i.e., cardiovascular diseases, dyslipidemia, oncological, pulmonary and autoimmune/immunological diseases) and the severity of COVID-19 itself. One exception is the level of education, which is significantly associated with the MoCA scores. This result is absolutely consistent with the most disparate literature on cognitive performance and the level of education and further corroborates our findings.

### Limitations

This study has certainly some limitations. First of all, we provided an “identikit” of the post-COVID-19 cognitive profile, analyzing the performance in the single subtests of the MoCA, a brief global screening test. We did not carry out a second-level assessment of each cognitive domain through more specific tests, which could have brought more conclusive results. However, because our participants underwent several examinations, a longer assessment could have been stressful and infeasible. Moreover, the MoCA has been proven to be a reliable and sensitive tool for the detection of post-COVID-19 cognitive sequelae [5]. Another limitation that we acknowledge is that the presence of potential psychiatric and neurological disorders, which may have influenced cognitive performance, was assessed through a semi-structured interview based on self-reports. While this approach provided valuable insights, more accurate and objective assessments could have been employed. However, constraints related to the emergency context and time limitations restricted our ability to adopt more comprehensive assessment methods. Furthermore, the sample size of the two groups considered, younger and older adults, was not balanced. This age bias may reflect the significantly higher rate of death due to COVID-19 in older adults, which must be taken in account. Lastly, this study lacked a control group composed of young and old individuals who had never been affected by the virus.

## 5. Conclusions

This paper contributes to increasing the knowledge about the existence, characterization, reversibility and age-related differences of COVID-19 cognitive sequelae. Overall, the main results suggest a continuous improvement in cognitive functioning over time for both younger and older adults, suggesting that the cognitive frailties often experienced after COVID-19 are at least partially reversible. Moreover, no significant differences (other than a physiological age-related difference) in the cross-sectional and longitudinal characterization of the cognitive profile emerged between younger and older adults. Thus, it is possible that the impact of COVID-19 on cognition may be similar across young and old adulthood in terms of the cognitive domains affected and their evolution over time. Lastly, the memory deficit observed primarily reflects a difficulty in attention and concentration that interferes mainly with retrieval processes. This result fits well with the concept of “brain fog” typical of post-COVID-19 syndrome and may also reflect the stress experienced while facing the pandemic. Importantly, ad hoc cognitive training could be implemented for the on-time treatment of cognitive frailties, which, if neglected, may progress to more significant difficulties in some individuals.

## Figures and Tables

**Table 1:** Descriptive characteristics of the sample as a whole and sorted by age.

	Total Sample (*n* = 202)	Age ≤ 65 (*n* = 144)	Age ≥ 65 (*n* = 58)
**Sociodemograpics and COVID-19-related** **clinical characteristics** **(Mean ± SD; *n* (%))**			
Age Biological sex (females)	56.788 ± 14.032 115 (56.9%)	50.243 ± 10.096 81 (56.3%)	73.115 ± 6.448 22 (37.9%)
Education Comorbidities Cardiovascular Pulmonary Autoimmune Dyslipidemia Oncological	12.573 ± 4.474 68 (33.7%) 34 (16.8%) 27 (13.4%) 58 (28.7%) 18 (8.9%)	13.483 ± 4.017 35 (24.3%) 21 (14.6%) 21 (14.6%) 35 (24.3%) 9 (6.3%)	10.948 ± 4.986 33 (56.9%) 13 (22.4%) 6 (10.3%) 23 (39.7%) 9 (15.5%)
Days from discharge to t0	54.600 ± 47.789	52.877 ± 47.966	59.786 ± 50.395
Days from COVID-19 swab COVID-19 severity AA Oxygen mask HFNC NIV IOT	79.000 ± 63.505 73 (36.1%) 80 (39.6%) 10 (4.6%) 7 (3.5%) 14 (6.9%)	75.936 ± 60.249 58 (40.3%) 52 (36.1%) 5 (3.5%) 6 (4.2%) 10 (6.9%)	86.226 ± 70.680 15 (25.9%) 28 (48.3%) 5 (8.6%) 1 (1.8%) 4 (6.9%)
**MoCA (Mean ± SD)**			
Total score	25.308 ± 3.419	26.388 ± 2.289	23.327 ± 4.435
Trial Making Test-B	0.892 ± 0.311	0.924 ± 0.267	0.818 ± 0.389
Copy cube	0.810 ± 0.393	0.915 ± 0.280	0.655 ± 0.480
Draw clock	2.713 ± 0.626	2.831 ± 0.495	2.491 ± 0.791
Naming	2.903 ± 0.400	2.941 ± 0.352	2.800 ± 0.524
Digit span	1.584 ± 0.630	1.700 ± 0.559	1.364 ± 0.704
Tapping	0.924 ± 0.266	0.950 ± 0.219	0.909 ± 0.290
Subtractions	2.817 ± 0.502	2.867 ± 0.429	2.782 ± 0.534
Repetition	1.569 ± 0.573	1.633 ± 0.501	1.455 ± 0.689
Fluency	0.624 ± 0.486	0.692 ± 0.464	0.509 ± 0.505
Abstraction	1.665 ± 0.571	1.775 ± 0.493	1.491 ± 0.605
Delayed recall	2.868 ± 1.559	3.225 ± 1.405	2.145 ± 1.693
Category cue	0.832 ± 0.949	0.697 ± 0.907	1.055 ± 1.061
Multiple choice cue	0.852 ± 1.015	0.765 ± 0.954	1.036 ± 1.154
Orientation	5.929 ± 0.277	5.950 ± 0.219	5.927 ± 0.262

Notes. AA: no oxygen supply; HFNC: high-flow nasal cannula; NIV: non-invasive ventilation; IOT: intensive oxygen therapy; MoCA: Montreal Cognitive Assessment.

**Table 2:** MoCA scores obtained at the three time points for the sample as a whole and sorted by age.

	T0		T1		T2	
	YA (*n = 47*)	OA (*n = 19*)	YA (*n = 47*)	OA (*n = 19*)	YA (*n = 47*)	OA (*n = 19*)
Total score	26.106 ± 2.139	21.737 ± 4.863	27.170 ± 2.316	23.526 ± 4.439	27.489 ± 2.094	23.842 ± 4.311
Trial Making Test-B	0.932 ± 0.255	0.684 ± 0.478	0.886 ± 0.321	0.737 ± 0.452	0.909 ± 0.291	0.526 ± 0.513
Copy cube	0.933 ± 0.252	0.579 ± 0.507	0.911 ± 0.288	0.421 ± 0.507	0.867 ± 0.344	0.421 ± 0.507
Draw clock	2.867 ± 0.588	2.316 ± 0.946	2.844 ± 0.475	2.263 ± 0.991	2.844 ± 0.424	2.421 ± 0.769
Naming	3.000 ± 0.365	2.684 ± 0.749	2.978 ± 0.147	2.842 ± 0.375	2.957 ± 0.206	2.737 ± 0.562
Digit span	1.681 ± 0.594	1.263 ± 0.653	1.596 ± 0.538	1.105 ± 0.737	1.596 ± 0.614	1.158 ± 0.834
Tapping	0.915 ± 0.282	0.842 ± 0.375	0.957 ± 0.204	0.947 ± 0.229	0.957 ± 0.204	0.947 ± 0.229
Subtractions	2.915 ± 0.282	2.737 ± 0.562	2.915 ± 0.282	2.737 ± 0.733	2.851 ± 0.551	2.684 ± 0.478
Repetition	1.638 ± 0.529	1.263 ± 0.733	1.596 ± 0.538	1.632 ± 0.597	1.681 ± 0.515	1.474 ± 0.697
Fluency	0.681 ± 0.471	0.474 ± 0.513	0.702 ± 0.462	0.579 ± 0.507	0.660 ± 0.479	0.684 ± 0.478
Abstraction	1.809 ± 0.398	1.421 ± 0.769	1.915 ± 0.282	1.684 ± 0.582	1.915 ± 0.282	1.684 ± 0.671
Delayed recall	2.979 ± 1.375	1.579 ± 1.427	4.021 ± 1.170	2.737 ± 1.661	4.298 ± 0.998	3.105 ± 1.449
Category cue	0.702 ± 0.931	1.474 ± 1.124	0.702 ± 0.907	0.789 ± 0.918	0.511 ± 0.748	0.684 ± 0.582
Multiple choice cue	0.872 ± 1.013	0.789 ± 1.134	0.255 ± 0.530	1.000 ± 1.202	0.191 ± 0.537	0.947 ± 1.079
Orientation	5.915 ± 0.282	5.895 ± 0.315	5.936 ± 0.247	5.842 ± 0.501	5.979 ± 0.146	6.000 ± 0.000

## Data Availability

The data presented in this study are available on request from the corresponding author due to the confidential nature of the data and the privacy of the participants.
